# An Effectively Uncoupled Gd_8_ Cluster Formed through Fixation of Atmospheric CO_2_ Showing Excellent Magnetocaloric Properties

**DOI:** 10.3390/ijms25010264

**Published:** 2023-12-23

**Authors:** Jonas Braun, Daniel Seufert, Christopher E. Anson, Jinkui Tang, Annie K. Powell

**Affiliations:** 1 Institute of Inorganic Chemistry (AOC), Karlsruhe Institute of Technology (KIT), Kaiserstr. 12, 76131 Karlsruhe, Germany; jonas.braun2@kit.edu (J.B.); daniel.seufert@kit.edu (D.S.); christopher.anson@kit.edu (C.E.A.); 2 Institute of Nanotechnology (INT), Karlsruhe Institute of Technology (KIT), Kaiserstr. 12, 76131 Karlsruhe, Germany; 3 Institute for Quantum Materials and Technologies (IQMT), Karlsruhe Institute of Technology (KIT), Kaiserstr. 12, 76131 Karlsruhe, Germany; 4 State Key Laboratory of Rare Earth Resource Utilization, Changchun Institute of Applied Chemistry, Chinese Academy of Sciences, Renmin Street 5625, Changchun 130022, China; tang@ciac.ac.cn

**Keywords:** Schiff base, coordination cluster, gadolinium, magnetocaloric effect

## Abstract

The [Gd_8_(opch)_8_(CO_3_)_4_(H_2_O)_8_]·4H_2_O·10MeCN coordination cluster (**1**) crystallises in $P\bar{1}$. The Gd_8_ core is held together by four bridging carbonates derived from atmospheric CO_2_ as well as the carboxyhydrazonyl oxygens of the 2-hydroxy-3-methoxybenzylidenepyrazine-2-carbohydrazide (H_2_opch) Schiff base ligands. The magnetic measurements show that the Gd^III^ ions are effectively uncoupled as seen from the low Weiss constant of 0.05 K needed to fit the inverse susceptibility to the Curie–Weiss law. Furthermore, the magnetisation data are consistent with the Brillouin function for eight independent Gd^III^ ions. These features lead to a magnetocaloric effect with a high efficiency which is 89% of the theoretical maximum value.

## 1. Introduction

In the last few decades, molecular systems have attracted considerable attention for their possible use as magnetic refrigerants via the magnetocaloric effect (MCE) [1,2,3]. This effect, discovered by Warburg [4], describes the heating or cooling of a material through the induction of entropy changes by an applied external magnetic field. Gd^III^ ions have been identified as optimal candidates for such clusters considering their large number of unpaired electrons and largely isotropic nature. This was shown in a Gd_7_ compound by Sharples et al., the first purely Gd^III^-containing cluster which was considered for magnetic refrigeration [3,5,6].

For the application of magnetic coolers, it is important that the field that needs to be applied in order to achieve the entropy change necessary is as low as possible [7,8]. This can be achieved when there are ferromagnetic interactions between the paramagnetic centres in the respective cluster [3]. 

If, however, the application of stronger fields is not an issue, another aspect becomes important, namely the efficiency of the MCE of the clusters. In order to achieve the highest possible efficiency of the MCE of a cluster, it is essential to minimise all magnetic exchange interactions between the paramagnetic centres. This leads to the entropy change observed experimentally coming close to the theoretically possible value which can be calculated using the following equation: $-\Delta S_{M}=n\;Rln\left(2S+1\right)/M_{w}$, where n is the number of ions, *R* is the universal gas constant, *S* is the spin of the ion (7/2 in the case of Gd^III^) and *M_w_* is the molecular weight of the cluster. This approach was recently validated by the publication of a Gd_12_Na_6_ cluster with uncoupled Gd^III^ ions (J = −0.01 K) reaching 99% of its maximum possible entropy change [9].

In terms of efficiency, the above-mentioned equation also implies that reducing the molecular weight of the diamagnetic part of the cluster is important. Therefore, using lighter organic ligands should be preferred over inorganic ligands such as tungstate-based polyoxometalates (POMs). Whilst these can show high absolute values of entropy change, the resulting low percentages of the observed entropy change in terms of the maximum possible are a result of the heavy metal inorganic POM ligands [10].

**Scheme 1:**
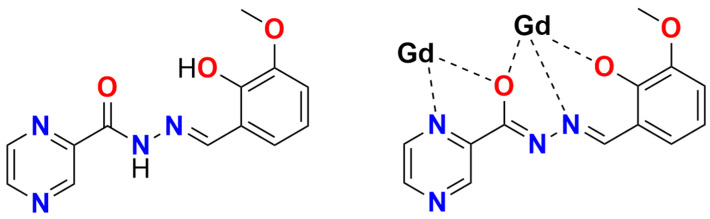
The neutral H_2_opch Schiff base ligand (**left**) and the coordination modes of the (opch)^2−^ ligands in (**1**) (**right**).

Schiff base ligands as a type of organic ligand with ample opportunity for modification and thus the possibility to easily fine tune a system, therefore provide an excellent opportunity to obtain lanthanide clusters which could show excellent magnetocaloric properties. The ligand used in this work, 2-hydroxy-3-methoxybenzylidenepyrazine-2-carbohydrazide (H_2_opch) (Scheme 1), in particular has been shown to be extremely versatile. Over the last decade H_2_opch was used to obtain lanthanide clusters with different nuclearities, from two to eight [11,12]. The ligand was also shown to be able to accommodate both 3d and 4f ions in the same cluster [12]. This tolerance towards ions of different sizes as well as the modifiability makes H_2_opch the ligand of choice for the work presented here in the search for new Gd^III^-based magnetic refrigerants.

## 2. Results and Discussion

### 2.1. Crystallography

**Figure 1:**
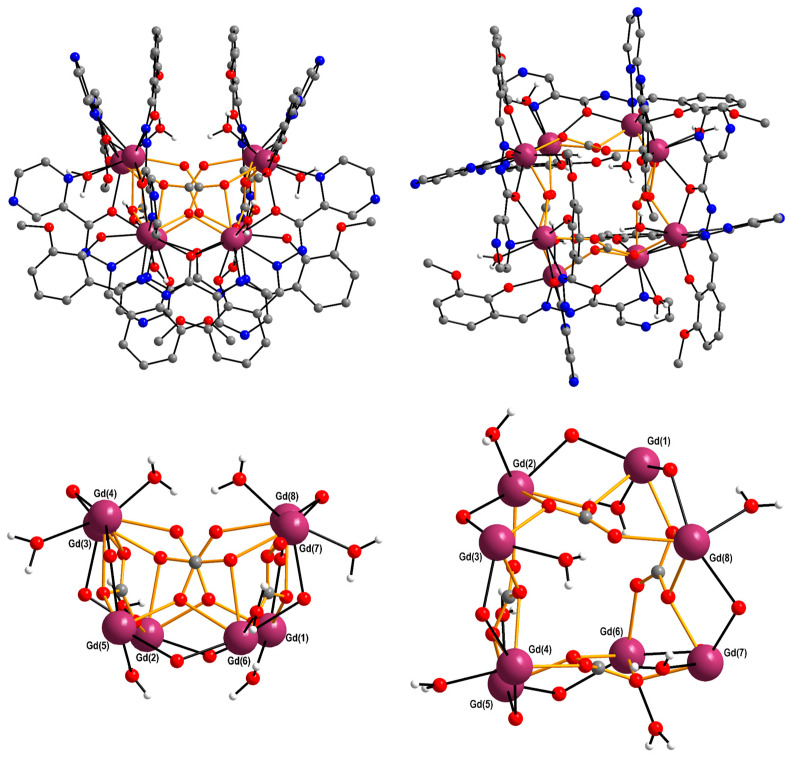
Two views of the molecular structure (**top**) and the core structure of (**1**) (**bottom**) highlighting the bridging modes of the carbonate ligands with orange bonds.

Here we report the synthesis, structure and magnetic properties of an octanuclear Gd_8_ cluster [Gd_8_(opch)_8_(CO_3_)_4_(H_2_O)_8_]·4H_2_O·10MeCN (**1**). Compound (**1**) crystallises in the triclinic space group $P\bar{1}$ with Z = 2. The core structure consists of eight Gd^III^ ions that are arranged in a distorted square antiprism and are held together by four carbonate ligands which are presumably derived from atmospheric CO_2_. The eight Gd^III^ ions are each chelated by a bridging opch^2-^ ligand, with the negatively charged carboxyhydrazonyl oxygen of each ligand bridging to a further Gd^III^ centre (see Scheme 1). The coordination sphere of each Gd^III^ ion is completed by one terminal water ligand (see Figure 1). The Gd⋯Gd distances are in the range 3.9534(5)–4.0203(5) Å. All the Gd-O-Gd angles of the direct oxygen bridges range from 107.2(2) to 115.2(2)°.

The molecular structure will not be described further, as it is essentially identical to that of a Dy_8_ cluster previously reported by some of us [11], which crystallised in *C*2/*c* with Z = 4. By contrast, compound (**1**) crystallises in the lower symmetry triclinic space group $P\bar{1}$ (see Table S1) which leads to differences in the packing. However, the two unit cells are related. Transformation of the unit cell of (**1**) by the matrix (1 1 0 1 −1 0 0 0 −1) gives a C-centred cell with *a* = 31.412, *b* = 18.519, *c* = 28.236 Å and α = 85.18, β = 107.64, γ = 91.19°, in good agreement with the reported unit cell parameters for the Dy_8_ apart from the deviations of α and γ from 90°. These differences seem to be steered by the choice of solvents used in the synthetic procedures (10 MeOH and 2 H_2_O as lattice solvent in the reported Dy_8_ compound compared to 10 MeCN and 4 H_2_O found in the lattice of (**1**)).

The stability of lanthanide–carbonate compounds is exemplified by the abundance of naturally occurring lanthanide–carbonate minerals [13] and the fixation of atmospheric CO_2_ has been reported for a number of lanthanide-based coordination complexes [14,15,16,17,18]. It was found that basic aqueous solutions are especially prone to fix atmospheric CO_2_ to form bicarbonate and carbonate ions in solution [16]. In the case of the synthesis of (**1**), no drying procedure was used on the solvents, which therefore contain water and the Gd(NO_3_)_3_·6H_2_O salt was used. Additionally, *N*-methyldiethanolamine (mdeaH_2_) was added, and this acts as a base creating favourable conditions for the absorption of CO_2_ to form bridging carbonate ligands.

### 2.2. Magnetic Properties

**Figure 2:**
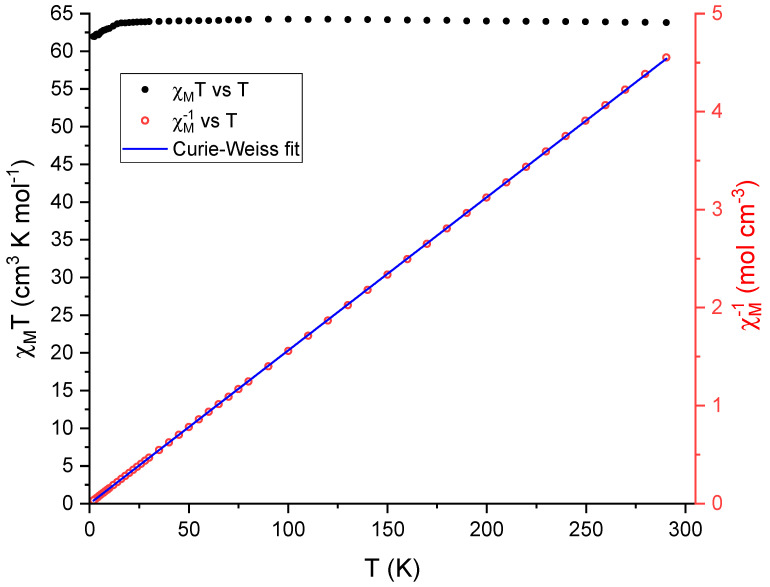
The χ_M_T vs. T plot (black dots), the inverse susceptibility vs. T plot (red circles) and the Curie–Weiss fit (blue line) for (**1**) between 300 and 2 K.

Temperature-dependent magnetic measurements using an applied dc field of 0.1 T were performed on (**1**) (Figure 2). At room temperature, the χ_M_T value of 63.80 cm^3^ Kmol^−1^ is in good agreement with the theoretical value of 63.04 cm^3^ Kmol^−1^ for eight uncoupled Gd^III^ ions. On lowering the temperature, the value of the χ_M_T product stays constant until ca. 25 K, below which a slight decrease was observed, reaching a minimum value of 61.96 cm^3^ Kmol^−1^ at 2 K. The extremely small decrease in χ_M_T of only 1.84 cm^3^ Kmol^−1^ over the measured temperature range is indicative of little to no coupling present between the Gd^III^ ions. This is further corroborated by the Curie–Weiss fit to the inverse susceptibility which results in an extremely small Weiss constant θ of 0.05 K and a Curie constant C of 63.8 cm^3^ Kmol^−1^.

This lack of interaction can be explained by the design of the ligand which has two pockets that make sure the Gd^III^ ions are well separated. In addition, this also reduces the overall molecular weight since it precludes the need for further ligands.

**Figure 3:**
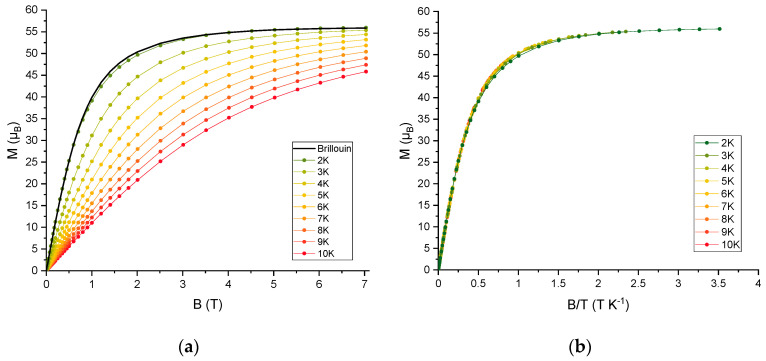
(**a**) Magnetisation and (**b**) reduced magnetisation curves of (**1**) at temperatures between 2 and 10 K and 0 and 7 T.

Isothermal magnetisation measurements were performed at temperatures between 2 and 10 K and magnetic fields between 0 and 7 T (see Figure 3, left). At 2 K, (**1**) reaches its saturation magnetisation of 55.97 µ_B_ at 7 T, which is very close to the theoretical value of 56 µ_B_ for eight S = 7/2 Gd^III^ ions. The lack of interaction between the Gd^III^ ions is further shown by the near perfect overlap of the magnetisation at 2 K with the Brillouin curve for eight S = 7/2 ions. In ac measurements, no out-of-phase signal could be observed, which is not surprising given the complete lack of anisotropy supported by the reduced magnetisation curves (Figure 3, right).

The negligible interaction between Gd^III^ ions suggested by the magnetic data presented above should be favourable for a strong magnetocaloric effect. Hence, the entropy change was calculated using the isothermal magnetisation measurements and Maxwell’s relation.

(1) $$\Delta S{\left(T\right)}_{\Delta H}=\int^{\;}{\left[\partial M\left(T,\;H\right)/\partial H\right]}_{H}dH$$

**Figure 4:**
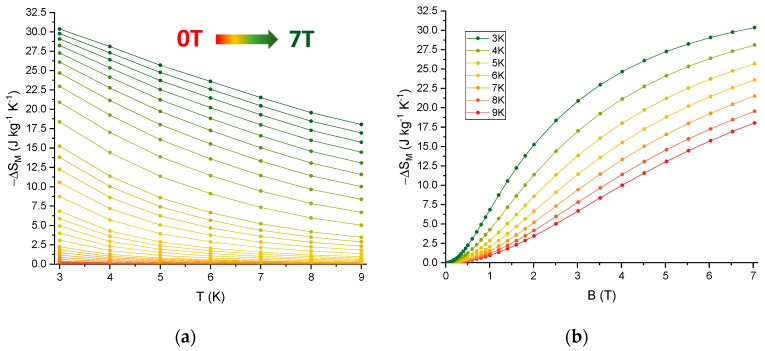
Molar entropy change calculated from isothermal magnetisation measurements vs. T (**a**) and B (**b**).

The temperature and field dependences are shown in Figure 4. The maximum experimental entropy change of 30.36 Jkg^−1^ K^−1^ is observed at 3 K and 7 T. This equates to 89% of the maximum possible entropy change of 34.10 Jkg^−1^ K^−1^ for this compound, placing (**1**) amongst the most efficient Gd_8_ compounds for magnetic refrigeration reported in the literature [19,20,21,22,23,24].

These six Gd_8_ clusters all show poorer MCE behaviour with efficiencies between 73% and 81% (further details can be found in Table 1). This appears to be a result of stronger coupling between the Gd^III^ ions as exemplified by the non-overlapping Brillouin and magnetisation curves in a [Gd_8_(HL)_6_(L)_2_(µ_3_–OH)_4_(µ_2_–OH)_2_(H_2_O)_4_] cluster [23]. A possible explanation for this could be the existence of direct hydroxo bridges to multiple other Gd^III^ ions in this compound (Gd1 in this compound is directly linked to six others). In addition, in the known compounds in the literature, the Gd^III^ ions are often interconnected by more than two oxygen-based bridges enhancing magnetic interactions. In compound (**1**), each Gd^III^ ion is only connected to two others by two bridges, one carbonate and the carboxyhydrazonyl oxygen of the Schiff base ligand, which both appear to provide less favourable superexchange pathways than, for example, hydroxo and oxo bridges. This is supported by the magnetic measurements presented above and the comparison with known compounds from the literature. Furthermore, the presence of bridging carbonate has previously been identified as a means to enhance MCE behaviour [20]. Therefore, the design of the ligand to avoid strong magnetic interactions and the use of anionic co-ligands such as carbonate (irrespective of whether the carbonate forms in situ by CO_2_ fixation or is added as a reagent) provide a general roadmap on how to improve MCE behaviour. Schiff bases lend themselves to the task of ligand design perfectly since they can be readily modified by variation of the amine or the aldehyde used.

## 3. Materials and Methods

The H_2_opch ligand was prepared using a procedure from the literature [26]. Gd(NO_3_)_3_·6H_2_O and *N*-methyldiethanolamine (mdeaH_2_) were obtained from commercial sources (Sigma Aldrich) and used as delivered.

**[Gd_8_(opch)_8_(CO_3_)_4_(H_2_O)_8_]·4H_2_O·10MeCN (1)**

H_2_opch (0.1 mmol) was dissolved in 5 mL of MeCN and mdeaH_2_ (0.3 mmol) was added. Gd(NO_3_)_3_·6H_2_O (0.1 mmol) was separately dissolved in 5 mL of MeCN and added dropwise to the ligand/base solution. The resulting yellow solution was stirred overnight, filtered and left undisturbed for slow evaporation. After two weeks, the product was obtained as red block crystals in a yield of 17.9 mg.

**IR (4000–400 cm^−1^):** 3388 (b, s), 3054 (m), 2996 (m), 2924 (m), 2831 (m), 1603 (s), 1591 (s), 1549 (s), 1530 (s), 1520 (s), 1474 (s), 1414 (s), 1389 (s), 1337 (s), 1237 (s), 1209 (s), 1180 (m), 1154 (s), 1104 (m), 1078 (m), 1059 (w), 1027 (m), 968 (w), 919 (m), 857 (m), 844 (m), 784 (w), 769 (w), 730 (m), 641 (w), 523 (w), 481 (w), 429 (w).

**C/H/N (calculated) corresponds to 10 lattice H_2_O:** C: 32.56%, H: 2.93%, N: 11.25%

**C/H/N (found)**: C: 32.32%, H: 2.548%, N: 11.49%.

Crystallography: A red single crystal of (**1**) with dimensions 0.16 × 0.14 × 0.12 mm^3^ was mounted on a Stoe StadiVari diffractometer equipped with a Mo GeniX 3D HF microfocus Mo-K_α_ source. Data were measured at 180 K, and corrected for absorption. Structure solution was by dual-space direct methods [27] and refinement by full-matrix least-squares refinement [28] against all data, on the Olex2 platform [29]. Non-H atoms were assigned anisotropic temperature factors. Geometric and rigid-bond restraints were applied to the lattice MeCN molecules as appropriate; the H-atoms of the lattice waters could not be located. Crystallographic data are summarised in Table S1.

The IR spectrum was measured on a Nicolet iS 50 with ATR attachment, the Elemental analysis was performed on a VarioELcube and magnetic SQUID measurements were performed on an MPMS-XL-7.

## 4. Conclusions

In conclusion we have shown the synthesis of a Gd_8_ cluster which forms via the fixation of atmospheric CO_2_ and is related to a previously reported Dy_8_ compound by Tang et al. [11]. As a result of the large Gd∙∙∙Gd distances, as well as the limited amount of superexchange interaction pathways compared with similar complexes in the literature, the Gd^III^ ions are essentially uncoupled in compound (**1**) [19,20,21,22,23,24]. This is apparent from the magnetic susceptibility measurements. The inverse susceptibility was fitted using the Curie–Weiss law, resulting in an extremely low Weiss constant of 0.05 K. Furthermore, the magnetisation data of (**1**) are consistent with a Brillouin function for eight S = 7/2 ions. This, and the complete lack of anisotropy, leads to the highly efficient MCE, which is 89% of the maximum possible value. This makes compound (**1**) one of the most efficient refrigerants reported using larger organic ligands. This also highlights the beauty of Schiff base ligands since they can be designed to serve a given purpose. To further improve MCE behaviour, Schiff base ligands can be designed to have multiple well-separated pockets to avoid strong magnetic coupling. Furthermore, it appears that the use of anionic co-ligands such as carbonate is to be preferred over hydroxo or oxo ligands.

## Figures and Tables

**Table 1:** Formulae, molecular weights and entropy changes with the efficiency in brackets, as well as the magnetic fields and temperatures the entropy change was measured at for different Gd_8_ compounds in the literature using larger organic ligands.

Compound	MW (g/mol)	−ΔS_M_ (J/kgK)	B (T)	T (K)	Reference
[Gd_8_(opch)_8_(CO_3_)_4_(H_2_O)_8_]·4H_2_O·10MeCN	4286.76	30.36 (89%)	7	3	this work
[Gd_8_(µ_3_-OH)_4_(L1)_4_(DEA)_4_Cl_4_](DMF)_2_(MeOH)	3404.00	31.4 (77%)	9	3	[19]
[Gd_8_(μ_3_-OH)_4_(CO_3_)_2_L_4_(PhCOO)_8_]	3982.60	28.38 (78%)	7	2	[20]
[Gd_8_(CH_2_OHCH_2_OH)_8_(SO_4_)_12_]·2(C_2_H_7_N)·2H_2_O	3012.30	36.86 (80%)	7	2	[21]
[Gd_8_(µ_3_-O)_4_(L)_8_(CH_3_COO)_4_(CO_3_)_2_]·15H_2_O	3463.85	32.49 (81%)	7	2	[22]
[Gd_8_(HL)_6_(L)_2_(µ_3_-OH)4(µ_2_-OH)_2_(H_2_O)_4_]	3777.88	25.5 (73%)	5	3	[23]
[Gd_8_(IN)_14_(μ_3_-OH)_8_(μ_2_-OH)_2_(H2O)_8_]·11H_2_O	3479.81	31.77 (80%)	7	2	[24]

Note: These values can be compared with complexes using smaller organic or inorganic ligands, such as [Gd_12_Na_6_(OAc)_25_(HCO_2_)_5_(CO_3_)_6_(H_2_O)_12_]·9H_2_O·0.5MeCN which is highly efficient (29.3 J/kgK (99%)) [9], and extended solids, such as [Gd(OH)CO_3_] which reaches extremely high entropy changes (66.4 J/kgK) [25].

## Data Availability

Full crystallographic data and details of the structural determination have been deposited with the Cambridge Crystallographic Data Centre as supplementary publication no. CCDC 2302991. Copies of the data can be obtained, free of charge, from https://www.ccdc.cam.ac.uk/structures/.

## Supplementary Materials

The following supporting information can be downloaded at: https://www.mdpi.com/article/10.3390/ijms25010264/s1.
