# Supplementary material for: An Effectively Uncoupled Gd8 Cluster Formed through Fixation of Atmospheric CO2 Showing Excellent Magnetocaloric Properties
Source: Int J Mol Sci. 2023 Dec 23;25(1):264. doi: 10.3390/ijms25010264 (PMC10779177; doi:10.3390/ijms25010264)
Supplement: Supplementary file 1 [file ijms-25-00264-s001.zip › ijms-2721260-supplementary.pdf]

An effectively uncoupled Gd_8 cluster formed through fixation of atmospheric CO_2 showing excellent magnetocaloric properties

Jonas Braun [1,2,3], Daniel Seufert [1], Christopher E. Anson [1], Jinkui Tang [4] and Annie K. Powell [1,2,3,*]

1) Institute of Inorganic Chemistry (AOC), Karlsruhe Institute of Technology (KIT), Kaiserstr. 12, 76131 Karlsruhe (Germany); jonas.braun2@kit.edu; daniel.seufert@kit.edu; christopher.anson@kit.edu; annie.powell@kit.edu

2) Institute of Nanotechnology (INT), Karlsruhe Institute of Technology (KIT), Kaiserstr. 12, 76131 Karlsruhe (Germany); jonas.braun2@kit.edu; annie.powell@kit.edu

3) Institute for Quantum Materials and Technologies (IQMT), Karlsruhe Institute of Technology (KIT Kaiserstr. 12, 76131 Karlsruhe (Germany); jonas.braun2@kit.edu; annie.powell@kit.edu

4) State Key Laboratory of Rare Earth Resource Utilization, Changchun Institute of Applied Chemistry, Chinese Academy of Sciences, Renmin Street 5625, Changchun 130022 (P.R. China); tang@ciac.ac.cn

* Correspondence: annie.powell@kit.edu

Crystallography

Table S1. Crystal data and structure refinement for (**1**).

Formula	$C_{128}H_{134}Gd_8N_{42}O_{48}$
Formula weight	4286.76
Crystal System	Triclinic
Space Group	$P\bar{1}$
a / Å	18.0662(6)
b / Å	18.3969(6)
c / Å	28.2356(10)
α / °	72.485(3)
β / °	77.273(3)
γ / °	61.037(2)
U / Å^3	7798.8(5)
Z	2
T / K	180
F(000)	4184
D_c / Mg m^{-3}	1.826
μ(Mo-Kα) / mm^{-1}	3.445
Data Measured	54635
Unique Data	29573
R_{int}	0.0428
Data with I ≥ 2σ(I)	19481
wR_2 (all data)	0.0972
S (all data)	0.969
R_1 [I≥2σ(I)]	0.0438
Parameters/Restraints	2099 / 291
Biggest diff. peak/hole / eÅ^{-3}	+1.889 / -1.207
CCDC number	2302991
